# Management of pediatric portal vein cavernous transformation: a seven-case single-center study

**DOI:** 10.3389/fped.2025.1627388

**Published:** 2025-08-07

**Authors:** Zhida Chen, Hui Liu, Wanfu Li, Gulimiremu Maimaiti, Ayiguzaili Maimaijiang, Yeliaman Jiayilawu, Aerxin Habuding, Runqi Xi, Haoyu Wang, Halimulati Huxitaer, FangJuan Song

**Affiliations:** First Affiliated Hospital, Xinjiang Medical University, Urumqi, Xinjiang, China

**Keywords:** cavernous transformation of the portal vein, portal vein cavernous transformation, liver transplantation, rex shunt, vascular reconstruction, pediatric

## Abstract

**Background:**

Cavernous transformation of the portal vein (CTPV) is one of the significant pathogenic factors of prehepatic portal hypertension in children. The Rex shunt, by reconstructing an intrahepatic portal venous pathway, not only effectively reduces portal pressure and restores physiological hepatic blood flow, but also promotes normal growth and development, making it the treatment of choice for CTPV. In contrast, traditional non-selective shunting procedures primarily alleviate symptoms of portal hypertension without restoring hepatic perfusion, thereby compromising growth potential. For patients unsuitable for the Rex shunt, living donor liver transplantation provides a definitive cure. Overall, both the Rex shunt and liver transplantation improve long-term outcomes in children with CTPV by reestablishing physiological portal circulation.

**Purpose:**

This study aims to summarize the clinical efficacy and institutional experience in the management of pediatric portal vein cavernous transformation.

**Methods:**

A retrospective analysis was conducted on seven children with portal vein cavernous transformation treated at the Department of Pediatric Surgery, First Affiliated Hospital of Xinjiang Medical University, between December 2021 and March 2025. The cohort included four boys and three girls, with ages ranging from 5 years and 10 months to 12 years. All patients had a history of esophagogastric variceal bleeding and hypersplenism. Preoperative evaluations included portal vein color Doppler ultrasonography, abdominal computed tomography angiography (CTA) to assess the portal venous system anatomy and blood flow dynamics. Following a rigorous assessment, six patients underwent living donor liver transplantation, and one patient underwent Rex shunt surgery.

**Results:**

All seven surgeries were successfully completed. During a follow-up period ranging from 3 to 42 months, no episodes of gastrointestinal bleeding were observed in any patient. Among the six patients who underwent liver transplantation, no cases of graft rejection, arterial complications, or biliary complications were reported. Postoperatively, all seven patients demonstrated a significant reduction in portal vein pressure and improvement in pancytopenia compared to preoperative values (*P* < 0.05).Of the six transplant recipients, three required portal vein reconstruction using allogeneic vascular grafts to establish continuity between the graft portal vein and the recipient superior mesenteric vein; in two cases, direct anastomosis was performed between the graft portal vein and a suitable segment of the recipient portal vein. The patient who underwent Rex shunt surgery received autologous inferior mesenteric vein grafting to reconstruct the portal pathway. Three transplant recipients developed portal vein anastomotic stenosis postoperatively, all of which were successfully managed with a single session of portal venography combined with balloon angioplasty.

**Conclusions:**

While the Rex shunt remains the gold standard for the treatment of portal vein cavernous transformation, living donor liver transplantation provides a viable alternative for patients unsuitable for Rex shunt reconstruction.

## 1 Introduction

Cavernous transformation of the portal vein refers to a compensatory pathological phenomenon in which a network of tortuous hepatopetal collateral veins develops following obstruction of the main trunk or branches of the prehepatic portal venous system due to congenital or acquired factors, accounting for approximately 40% of prehepatic portal hypertension cases in children (1). The condition is named for its characteristic sponge-like appearance on gross anatomy (2). CTPV can lead to secondary portal hypertension, with clinical manifestations including gastrointestinal bleeding, splenomegaly with hypersplenism, and ascites. In pediatric patients, the initial presentation often involves upper gastrointestinal bleeding due to rupture of esophagogastric varices, which remains the leading cause of mortality, with an estimated 10% of children with CTPV dying from upper gastrointestinal hemorrhage (3).

With advancements in pediatric surgery and liver transplantation techniques (4), current surgical treatments for pediatric CTPV include devascularization procedures, portosystemic shunting, Rex shunt, and liver transplantation; however, no standardized international guidelines have been established. Due to the ongoing growth and developmental needs of children, therapeutic strategies focus not only on reducing portal pressure but also on restoring physiological portal venous perfusion, which significantly differs from adult treatment approaches. Both Rex shunt and liver transplantation, by reconstructing the portal venous system, can effectively restore hepatic blood flow, alleviate symptoms of portal hypertension, and support normal growth and development in affected children.

This study retrospectively analyzed seven pediatric CTPV cases treated at the First Affiliated Hospital of Xinjiang Medical University between December 2021 and March 2025. By systematically collecting perioperative clinical data, we aim to summarize the clinical characteristics and management strategies for CTPV in children, thereby contributing to the standardization of its diagnosis and treatment.

## 2 Methods

### 2.1 Patient characteristics and clinical data

A retrospective review was conducted of seven pediatric patients with portal vein cavernous transformation (PVCT) treated at the Department of Pediatric General Surgery, First Affiliated Hospital of Xinjiang Medical University between December 2021 and March 2025. Six patients underwent living donor liver transplantation (LDLT). The graft types included four left hemi-livers (with the middle hepatic vein), one right hemi-liver (without the middle hepatic vein), and one left lateral segment. Allogeneic vascular grafts were used in three of these cases to reconstruct the portal vein. Donor selection strictly adhered to national legal and ethical regulations and was entirely voluntary. Comprehensive evaluations were performed, focusing on donor age, laboratory tests, imaging findings, hepatic vascular anatomy, and ABO blood group compatibility. One patient underwent a Meso-Rex bypass procedure using the autologous inferior mesenteric vein as the interposition graft. The study was approved by the Ethics Committee of the First Affiliated Hospital of Xinjiang Medical University, and written informed consent was obtained from all patients and their legal guardians.

### 2.2 Perioperative management

**Figure 1:**
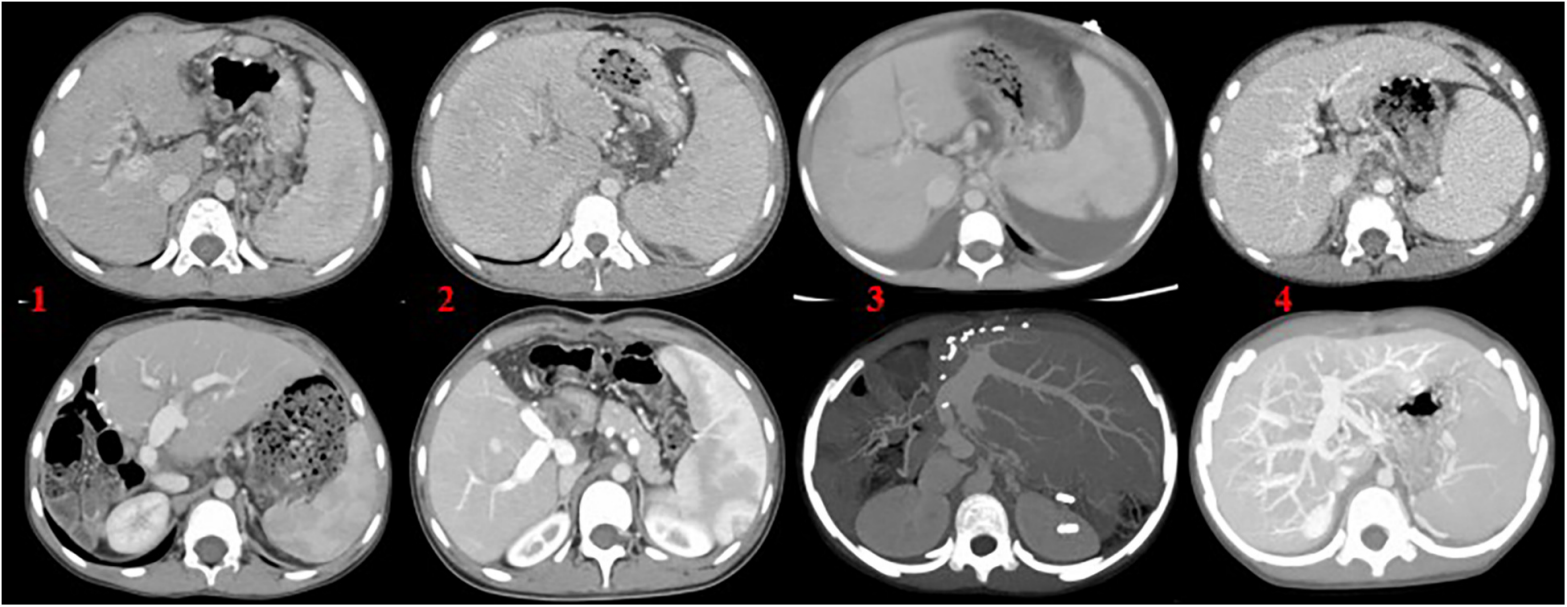
**(1)** Left lobe liver transplantation with marked splenomegaly; **(2)** right lobe liver transplantation with the use of an allogeneic vascular graft, accompanied by splenomegaly; **(3)** left lateral segment transplantation, with splenomegaly and ascites; **(4)** Rex shunt surgery utilizing the autologous inferior mesenteric vein. The upper images represent preoperative findings, while the lower images show postoperative outcomes.

**Figure 2:**
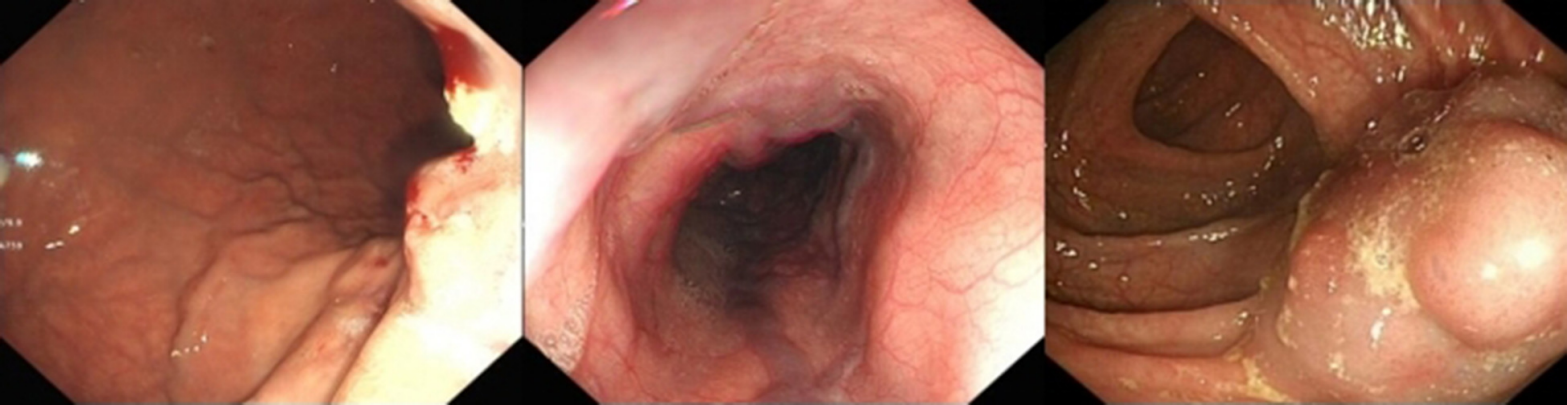
Endoscopic findings endoscopic images showing esophagogastric varices and submucosal tortuous vascular clusters in the intestine.

(1) Preoperative ultrasonography was performed to evaluate liver parenchymal atrophy, splenomegaly, and the presence of ascites. Doppler ultrasound of the hepatic vasculature was used to measure the sagittal diameter and flow velocity of the intrahepatic portal vein.
(2) Abdominal computed tomography angiography (CTA) was employed to evaluate the anatomical status of the portal vein tributaries and the intrahepatic right and left branches. In five patients (Cases 2, 3, 4, 5, and 6), preoperative CTA demonstrated either occlusion or hypoplasia of the left portal vein branch. Two patients also presented with intrahepatic bile duct dilation. Case 1 had previously undergone a Rex shunt, but the symptoms of portal hypertension persisted postoperatively. CTA revealed occlusion of both the left portal vein and the bypass conduit. In Case 4, CTA showed left portal vein occlusion accompanied by ascites. In Case 5, a slender left portal vein was observed along with massive splenomegaly and splenic infarction (Figure 1). In Case 6, the left portal vein was hypoplastic, and no normal portal venous structure was visualized in the Rex recess.
(3) Esophagogastroduodenoscopy (EGD) was performed to assess the degree of esophageal and gastric varices (Figure 2).

Based on comprehensive evaluations, six patients were deemed unsuitable for initial or repeat Rex shunt surgery, and only one patient met the criteria for Rex shunt.

Preoperative coagulation profiles were assessed in all seven patients. Mild coagulopathy was detected in some cases (Table 1). The postoperative anticoagulation regimen included monitoring of INR; continuous heparin infusion was initiated when INR was <1.5. Warfarin was started one week postoperatively, targeting an INR range of 1.5–2.0. Once the therapeutic range was achieved, heparin was discontinued, and warfarin was maintained for six months post-transplant.

**Table 1:** Preoperative coagulation profiles.

Case no.	PT (s)	APTT (s)	Fibriogen (g/L)	D-dimer (ng/L)	INR	FDP (ug/ml)
1	12.8	30.3	2.66	191.0	2.30↑	1.09
2	13.7	34.2	1.72↓	132.0	1.21↑	0.35↓
3	12.8	29.7	3.22	81.0	1.08	0.95↓
4	11.6	24.9	2.36	1,927.0↑	1.01	9.32
5	18.1↑	30.0	1.35↓	502.0↑	1.62↑	2.90
6	17.9↑	29.1	3.26	251.0↑	1.52↑	1.61
7	12.0	29.1	2.28	191.0	1.04	4.29

### 2.3 Portal venous reconstruction

#### 2.3.1 Living donor liver transplantation

(1) Donor Procedure: During graft procurement, the portal vein was divided as close as possible to the bifurcation of the left and right branches to maximize graft vessel length. (2) Recipient Procedure: Careful dissection and mobilization of the hepatic hilum were performed. In children with CTPV, the normal portal vein structure is typically absent at the hepatic hilum, with numerous dilated collateral veins exhibiting thin, fragile vessel walls. Intraoperative injury to these vessels could result in uncontrollable bleeding; therefore, meticulous surgical technique was essential. Portal venous pressure was measured intraoperatively by puncturing a suitable collateral vein. In all five liver transplant recipients, dissection was extended to the confluence of the superior mesenteric vein (SMV) and splenic vein. In three cases, intraoperative evaluation determined that direct end-to-end anastomosis between the graft portal vein and the recipient SMV was not feasible; thus, allogeneic vascular grafts were used for bridging reconstruction. In the remaining two cases, direct tension-free end-to-end anastomosis was achieved between the graft portal vein and the SMV-splenic vein confluence.

#### 2.3.2 Rex shunt surgery

The patient was placed in the supine position, and a midline vertical incision approximately 10 cm in length was made below the xiphoid process. Careful dissection around the porta hepatis revealed numerous tortuous vessels, which were intraoperatively confirmed to represent cavernous transformation of the portal vein. The sagittal portion of the liver tissue was dissected, and the left portal vein branch was identified and mobilized. Vascular clamps were applied, and a longitudinal incision was made along the sagittal portion of the liver.

**Figure 3:**
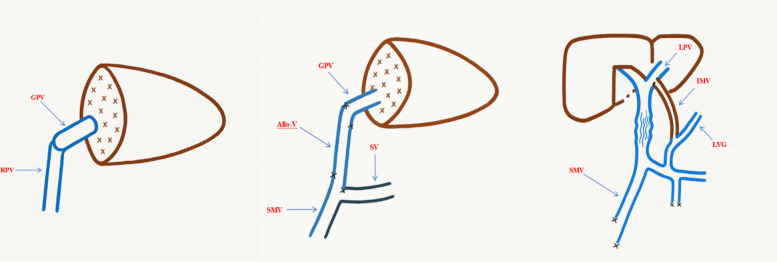
Schematic diagram of vascular reconstruction during surgery. GPV, graft portal vein; RPV, recipient portal vein; allo-V, allogeneic vascular graft; SV, splenic vein; LPV, left portal vein branch; IMV, inferior mesenteric vein; LVG, left gastric vein.

The inferior mesenteric vein (IMV) was subsequently located, fully exposed, and mobilized for approximately 8 cm for use as a bridging vessel. The IMV was routed anterior to the pancreas and posterior to the stomach to create a retrogastric tunnel. End-to-side anastomoses were performed between the bridging IMV and the left portal vein branch, as well as between the IMV and the coronary vein of the stomach. Portal venous pressures were measured at the superior mesenteric vein (SMV) both before and after the anastomoses (Figure 3).

## Results

Among the seven pediatric patients with portal vein cavernous transformation, there were four males and three females, with ages ranging from 5.8 to 12 years (median age: 8.4 years). Basic demographic and clinical information of the patients is summarized in Table 2.

**Table 2:** Clinical characteristics.

Case no.	Sex	Age at surgery (years)	Clinical presentation	History of previous surgery	Surgical procedure	Bridging vessel	Operative time (h)	Intraoperative blood loss (ml)
1	Male	12.0	Recurrent hematemesis and melena	Splenectomy, Rex shunt, multiple endoscopic ligations	Living donor liver transplantation (left hemiliver)	None	13.60	1,900
2	Male	7.0	Hematemesis, fatigue, melena, hypersplenism	None	Living donor liver transplantation (left hemiliver)	None	11.20	300
3	Female	11.0	Hematemesis, melena, fatigue, learning difficulties	Endoscopic variceal ligation	Living donor liver transplantation (left hemiliver)	None	13.67	1,700
4	Female	13.0	Hematemesis, melena, hypersplenism, epistaxis	Endoscopic variceal ligation	Living donor liver transplantation (right hemiliver)	Allogeneic vein graft (iliac vein)	13.17	500
5	Male	5.8	Hematemesis, melena, splenomegaly, gingival bleeding	Endoscopic variceal ligation	Living donor liver transplantation (left lateral segment)	Allogeneic vein graft (iliac vein)	9.98	600
6	Female	13.0	Hematemesis and melena	Endoscopic variceal ligation	Living donor liver transplantation (left hemiliver)	Allogeneic vein graft (iliac vein)	10.08	800
7	Male	5.9	Recurrent hematemesis and melena	Endoscopic variceal ligation	Rex shunt surgery	Autologous inferior mesenteric vein	5.00	150

Among the six patients who underwent living donor liver transplantation (LDLT), the mean operative time was (11.95 ± 1.74) hours. The graft-to-recipient weight ratio (GRWR) was (1.20 ± 0.43)%. The mean anhepatic phase duration was (71.67 ± 22.26) minutes, warm ischemia time was (2.50 ± 1.87) minutes, and cold ischemia time was (110.33 ± 54.25) minutes. The mean intraoperative blood loss was (966.67 ± 668.33) ml.

The patient who underwent Rex shunt surgery had an operative time of 5 h and an intraoperative blood loss of 150 ml.

**Figure 4:**
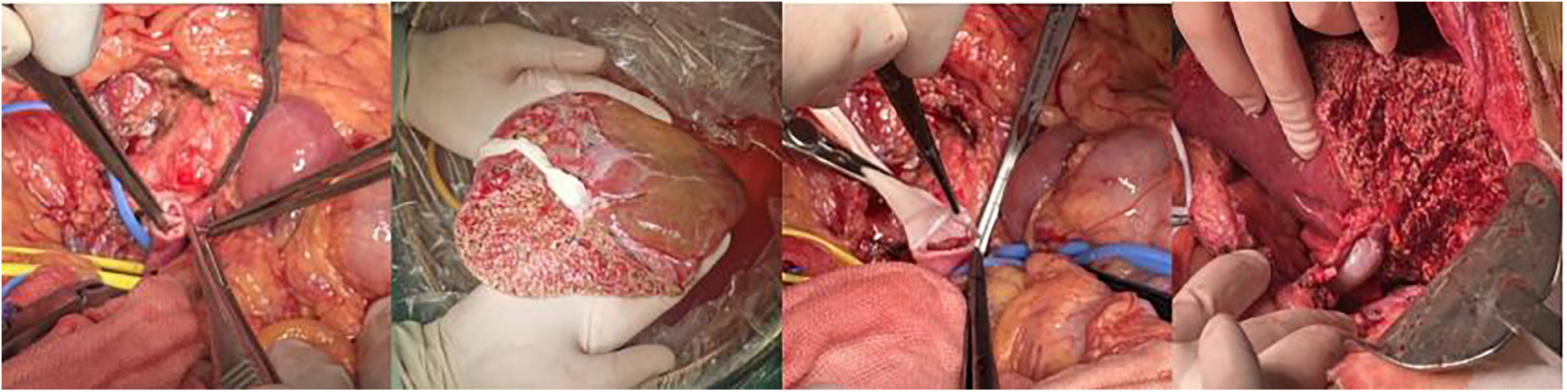
Intraoperative conditions **(1)** portal vein thrombosis; **(2)** graft and bypass vessels; **(3)** anastomosis between the graft and recipient portal vein; **(4)** filling of bypass vessels after portal vein opening.

Intraoperative portal vein pressure measurements performed before abdominal closure demonstrated significant reductions compared to preoperative levels. At three months postoperatively, hematologic parameters showed significant improvements in all patients, with pancytopenia markedly corrected compared to preoperative values (*P* < 0.05) (Table 3). Intraoperative liver appearance is shown in (Figure 4).

**Table 3:** Comparison of blood cells and portal pulse pressure.

Parameter	Preoperative value	Postoperative value	*T*-value	*P*-value
White blood cell count (×10^9^/L)	2.00 ± 0.47	6.49 ± 1.74	−6.582	<0.001
Hemoglobin (g/L)	60.43 ± 15.11	115.43 ± 8.98	−8.279	<0.001
Platelet count (×10^9^/L)	55.00 ± 24.06	158.86 ± 58.63	−4.336	<0.001
Superior mesenteric vein pressure (cmH₂O)	34.30 ± 6.48	21.44 ± 5.87	3.892	0.002

**Figure 5:**
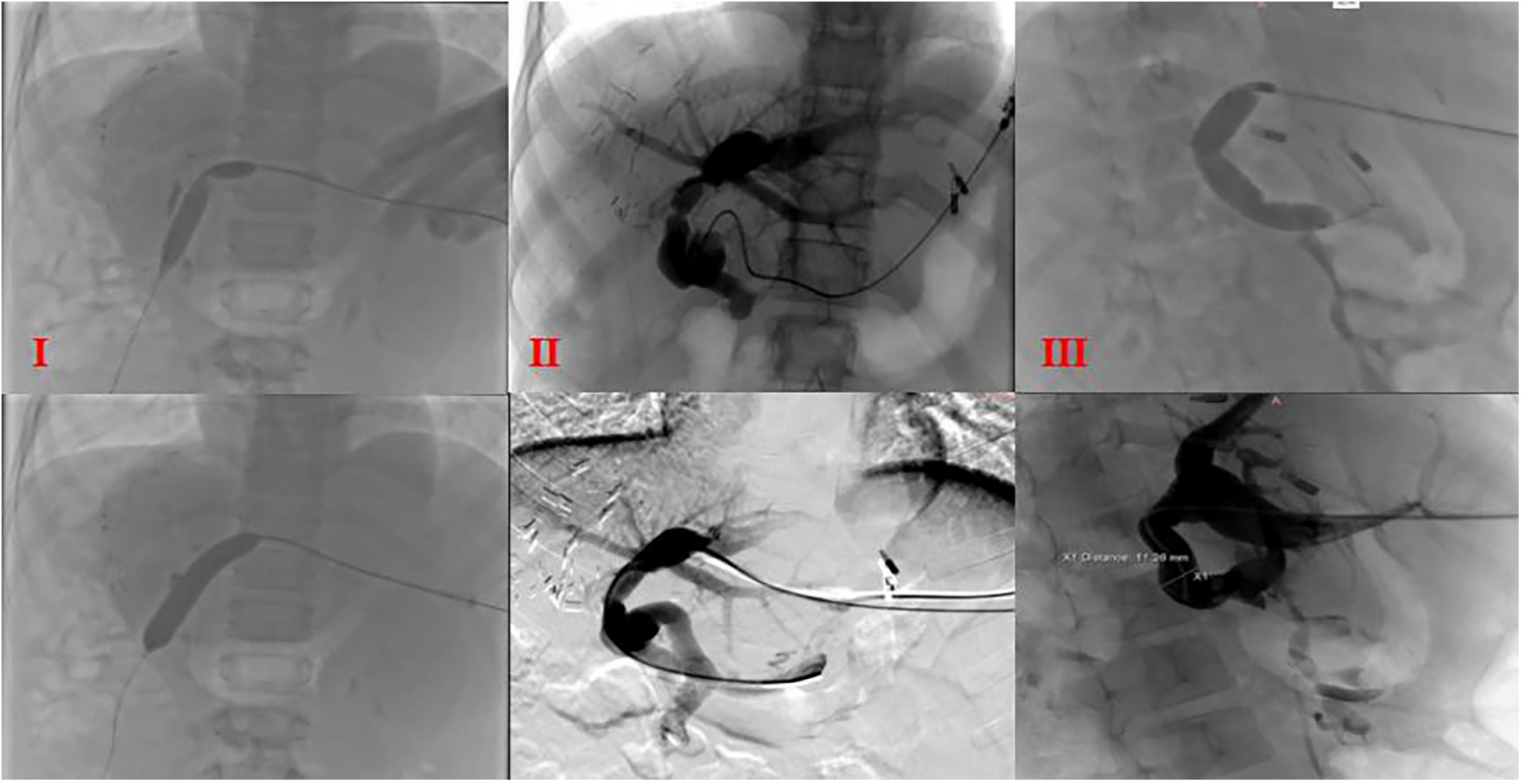
Portal venography and balloon dilation postoperative balloon dilation and venography in cases **(I)**, **(II)**, and **(III)**; I: portal venography showing angulation at the anastomotic site; II and III: stenosis at two anastomotic sites.

Three liver transplant recipients (Cases 2, 3, and 5) developed portal vein anastomotic stenosis. In Case 5, a suspicious thrombus was observed within the bridging graft. The portal venous imaging for the remaining cases demonstrated satisfactory graft portal vein patency. All three patients with anastomotic stenosis were successfully treated with portal venography combined with balloon angioplasty (Figure 5).

During a follow-up period of 3–42 months, all seven patients showed favorable outcomes. No episodes of graft rejection, vascular complications, or biliary complications were observed. Portal vein pressures remained significantly reduced, and no cases of gastrointestinal bleeding, epistaxis, or gingival bleeding occurred during the follow-up period. In this cohort, none of the six liver transplant recipients experienced acute rejection, and no CMV or EBV infections were observed during the follow-up period.

## Discussion

Cavernous transformation of the portal vein (CTPV) primarily results from partial or complete obstruction of the main portal vein or its branches, leading to elevated local venous pressure and remodeling of the surrounding venous network, which appears as a characteristic “cavernous” change on imaging. Although the exact etiology of CTPV remains unclear, contributing factors may include postnatal umbilical infections, mesenteric vascular or abdominal inflammatory processes, and congenital portal vein malformations (5). Most children with CTPV are asymptomatic in the early stages and are often diagnosed during routine examinations prompted by findings of hypersplenism or pancytopenia. As the disease progresses, patients may develop severe complications such as esophageal varices and gastrointestinal hemorrhage. Imaging studies play a pivotal role in the diagnosis of CTPV. Abdominal ultrasonography, as a noninvasive and sensitive screening tool, can assess portal vein diameter, blood flow velocity, and flow direction (6). If abnormalities are detected, further abdominal computed tomography angiography (CTA) can provide detailed visualization of the main portal vein and its branches. However, CTA has certain limitations in evaluating the patency of the Rex recess. On one hand, the presence of the left portal vein on imaging does not guarantee a functional hepatopetal flow pathway. In some cases, although the left portal branch is visualized on CTA, intraoperative exploration may reveal atrophy or obliteration of the Rex recess, rendering the Meso-Rex bypass unfeasible. On the other hand, CTA cannot assess the direction of blood flow, making it difficult to determine whether hepatopetal portal flow is preserved. Therefore, when imaging findings, clinical presentation, and liver function do not allow for a conclusive assessment, retrograde portography is recommended to determine Rex recess patency. However, due to technical constraints and the lack of pediatric-specific equipment, retrograde portography is not routinely performed at our center. Consequently, this study primarily relied on non-invasive imaging modalities such as CTA and ultrasonography to assess the development and patency of the Rex recess. Although limitations exist, we aimed to integrate multimodal imaging findings with intraoperative observations to ensure accuracy in surgical decision-making. Moreover, intraoperative findings confirmed atrophy of the Rex recess and the absence of functional blood flow in the left portal vein branch.

Treatment strategies for pediatric CTPV must comprehensively consider the extent of portal vein involvement, hepatic perfusion status, and the growth and developmental needs of the child. Although traditional portosystemic shunt procedures can partially relieve portal hypertension, they do not adequately restore hepatic perfusion and may impair growth, leading to their gradual abandonment. In certain cases, selective shunting may be considered as a second option when Rex shunt is not feasible, especially Warren's shunt. However, it does not address the underlying pathology of the portal venous system, nor does it fully restore portal blood flow. Over the long term, it may lead to hepatic dysfunction, portal hypertensive cholangiopathy, and hepatopulmonary syndrome (7, 8). Consequently, clinical focus in recent years has shifted toward Rex shunt surgery and liver transplantation. The Rex shunt aims to reestablish physiological hepatic blood flow by constructing an extrahepatic portal venous bypass (9), thereby not only curing portal hypertension but also promoting normal growth and development. Multiple clinical studies have demonstrated favorable short- and mid-term outcomes following Rex shunt surgery, supporting its role as the preferred option for pediatric CTPV. However, not all children are suitable candidates for Rex shunt (10). Based on our center's experience, the following criteria should be met: (1) a patent left intrahepatic portal vein branch with a diameter of at least 3 mm; (2) an available bridging vessel with a diameter of at least 5 mm; and (3) patent right and left intrahepatic portal vein branches without concomitant liver diseases such as cirrhosis or hepatitis (11). Therefore, precise preoperative imaging evaluation is essential for patient selection. The classic Meso-Rex shunt, first described by Jean de Ville de Goyet (12), involves autologous internal jugular vein grafting to connect the superior mesenteric vein to the left portal vein (Rex recess), thereby restoring hepatopetal portal flow. While this technique is still widely regarded as the standard approach, its practical application—particularly in pediatric patients—may be limited by challenges in harvesting the internal jugular vein, anatomical variations, and technical constraints. In response to these challenges, our center has routinely adopted a modified Meso-Rex technique tailored to pediatric anatomical characteristics. In this approach, the inferior mesenteric vein is used as an autologous graft, which is tunneled anterior to the pancreas and posterior to the stomach, and anastomosed end-to-side with both the left portal vein and the left gastric vein. This technique avoids cervical dissection, simplifies the procedure, and has demonstrated favorable safety and feasibility in children with marked cavernous transformation of the portal vein.

For children who are not suitable candidates for Rex shunt surgery, our center recommends living donor liver transplantation (LDLT). In recent years, advancements in surgical techniques and optimization of immunosuppressive regimens have led to five-year survival rates exceeding 90% in pediatric liver transplantation (13, 14). LDLT offers several unique advantages in the treatment of pediatric CTPV, including a stable graft supply, greater control over the timing of surgery, shorter cold ischemia times, and a lower incidence of both acute and chronic rejection episodes.

One of the main technical challenges in liver transplantation for pediatric CTPV is portal vein reconstruction. Based on our center's experience, the following strategies are critical: (1) During graft procurement from the living donor, the portal vein should be transected as close as possible to the bifurcation of the left and right branches to maximize the graft portal vein length; (2) In the recipient, meticulous dissection of the portal vein and its tributaries is necessary, with thorough exploration of the portal vein trunk, superior mesenteric vein (SMV), and splenic vein to assess for thrombosis; (3) Direct end-to-end anastomosis between the recipient portal vein trunk and the graft portal vein should be performed at a tension-free site, avoiding the use of bridging vessels whenever possible; (4) If bridging vessels are required, the recipient's dilated coronary vein of the stomach or splenic vein remnants should be prioritized. If these vessels are unsuitable, pre-prepared allogeneic grafts should be used. Bridging grafts should have a diameter greater than 5 mm, minimal branching, and sufficient length to ensure a tension-free anastomosis. Commonly used vessels include the internal jugular vein or iliac vein, and attention must be paid to the directionality of venous valves during anastomosis; (5) Correct axial alignment between the graft portal vein and the recipient-side portal vein must be ensured at the anastomosis. In the graft, portal clamps can assist in determining the proper axis. In the recipient, temporary opening of the portal vein stump to allow blood flow can help confirm the natural axial alignment; (6) The recipient portal vein stump should be tailored into a laterally slanted teardrop-shaped opening based on the type of graft. This configuration facilitates a natural angle post-anastomosis, minimizing the risks of portal vein redundancy, angulation, kinking, and stenosis, and reduces the likelihood of future portal vein distortion as the graft liver grows; (7) Adequate growth factor (redundancy) should be preserved at the anastomotic site to avoid stenosis after portal vein reperfusion; (8) Before abdominal closure, the graft should be properly fixed, and portal vein morphology reassessed to ensure there is no redundancy, torsion, or angulation. Intraoperative Doppler ultrasound should be used to evaluate the anastomotic diameter, inner wall smoothness, blood flow direction and velocity, presence of angulation, and detection of turbulent flow. Portal venous pressure should also be measured by direct puncture.

Regardless of the treatment modality employed, long-term and effective anticoagulation therapy is essential to prevent portal vein thrombosis (14). The most common postoperative complication is portal vein anastomotic stenosis. Early diagnosis primarily relies on Doppler ultrasonography and portal venography. Timely interventions, such as balloon angioplasty, are crucial for improving long-term outcomes. Portal vein angiography is the gold standard for diagnosing portal vein stenosis after liver transplantation. If the diameter of the portal vein anastomosis is less than 50% of the adjacent normal portal vein diameter and the pressure gradient exceeds 5 mmHg, portal vein stenosis (PSV) is diagnosed (15). Balloon angioplasty can be performed three weeks after surgery. Postoperatively, anticoagulation therapy is required, and warfarin is continued for six months after the angioplasty. In this study, three patients underwent portal venography followed by balloon angioplasty once, resulting in satisfactory therapeutic outcomes. For patients with persistent stenosis despite multiple balloon angioplasties, some researchers have considered the possibility of portal vein stent placement. However, others caution against stent placement in children, as stents do not grow with the child, potentially altering portal blood flow (16). In some cases, stent placement may necessitate subsequent liver transplantation. Moreover, the impact of preoperative coagulation abnormalities on the incidence of postoperative portal vein thrombosis remains to be fully elucidated. Studies have shown that inherited thrombophilic disorders—such as Factor V Leiden mutation and deficiencies in protein C and protein S—are associated with venous thromboembolism in children. This highlights the importance of screening for hereditary thrombophilia, particularly in patients with a personal or family history of thrombosis. Early identification of thrombotic risk may facilitate timely intervention and help reduce the incidence of postoperative portal vein thrombosis (17).

In conclusion, comprehensive management of pediatric CTPV should be based on early screening, precise imaging evaluation, and individualized surgical strategies. For patients meeting the criteria for Rex shunt surgery, restoration of physiological hepatic blood flow through Rex shunting can promote normal growth and development. For those unsuitable for Rex shunt or with failed prior Rex surgery, liver transplantation provides an effective curative option. Future collaborative efforts among global centers are encouraged to share clinical experiences, establish standardized diagnostic and treatment guidelines, and ultimately improve therapeutic outcomes and long-term prognoses for children with CTPV.
